# Assessing shared decision-making: a case study of third-year medical student standardized patient encounters

**DOI:** 10.5116/ijme.676f.d093

**Published:** 2025-01-22

**Authors:** Vikranth Induru, Catherine Deffendall, Ceceila Theobald, Jennifer Green, Heather Ridinger

**Affiliations:** 1 Lewis Katz School of Medicine at Temple University, USA; 2 Department of Rheumatology, Vanderbilt University Medical Center, USA; 3 Department of Medicine, Vanderbilt University Medical Center, USA; 4 Department of Medicine and Pediatrics, Vanderbilt University Medical Center, USA

**Keywords:** Shared-decision making, communication, empathy, standardized patient encounter, skill assessment

## Abstract

**Objectives:**

We aimed to determine if shared decision-making (SDM) self-assessment of a standardized patient (SP) scenario was reliable, specifically whether students’ communication resulted in each SP-student pair reporting internally consistent final treatment choices. We hypothesized student self-assessment would differ from SP and faculty assessment indicating a need for multisource feedback.

**Methods:**

In this observational case study from 2016-2017, all third-year post-clerkship medical students received evidence-based treatment options for sinusitis and SDM lectures followed by a SP encounter on sinusitis. Students, faculty, and SPs then completed a 9-question assessment covering SDM skills, perceived empathy, and final treatment choice. Mean self-assessment was compared to faculty and SP scores using paired t-test. Effectiveness of SDM communication was assessed as rate of treatment agreement, defined as percent of student-SP pairs reporting consistent final treatment choices.

**Results:**

Compared to SPs (M = 23.4, SD = 3.6), 120 students (M = 22.6, SD = 3.1) reported lower mean SDM skills, t_(119)_ = 2.25, p = .027. Conversely, SPs (M = 8.0, SD = 1.5) compared to students (M = 8.5, SD = 1.1) reported lower mean empathy, t_(119) _= 3.43, p < .001.  Faculty ratings of students’ SDM (M = 22.7, SD = 3.5) and empathy (M = 8.3, SD = 1.7) was not statistically different than students’ ratings, t_(119)_ = 0.46, p = .645 and t_(119)_ = 1.40, p = .164 respectively. Seventeen (14%) student-SP pairs reported different final treatment choices.

**Conclusions:**

We demonstrated the limitations of self-perception of SDM and empathy skills, highlighting the importance of multisource feedback for assessing trainee communication skills. Disagreement between student-SP pairs on perceived final treatment choice underscores the need for ongoing SDM practice.

## Introduction

Multiple studies have examined how effective physician communication can improve patient adherence, emotional health, and functional status.^1^^-^^4^ Shared decision-making (SDM) is a patient-centered communication technique based on theories of self-determination and relational autonomy that involves collaboratively developing a treatment plan based on patient values and preferences.^5^^-^^7^ SDM skills are increasingly cited as a critical part of a physician’s skillset and implemented in undergraduate medical education (UME) curricula to improve medical student competency in SDM.^8^  The Association of American Medical Colleges (AAMC), Accreditation Council for Graduate Medical Education (ACGME), and the British National Institute for Health and Care Excellence (NICE) all have position statements emphasizing the importance of shared decision-making.^9^^-^^11^

Not only are SDM skills central to providing patient-centered care, SDM communication strategies are especially helpful in collaboratively making treatment choices when there are two equally appropriate treatment options. In these instances, physicians can use patient-centered communication skills to summarize the evidence and guide joint decision-making with the patient rather than relying solely on clinician judgement, habitual practice patterns or personal preferences.^12^ It is critical that medical trainees can effectively convey the potential risks and benefits associated with treatment options in an empathetic and patient-centered way to collaboratively make a treatment choice based on patient preference. Effective communication strategies have been associated with improved patient knowledge and satisfaction with treatment decisions in diabetes care.^13^ In cardiac literature, SDM has also been associated with decreased decisional conflict and personal uncertainty about which course of action to take when competing options are available and involve potential risks.^14^^, ^^15^

Efforts to incorporate SDM into health professions education have traditionally cited key barriers which include research evidence of its effectiveness, lack of faculty training and clinical champions, availability of decision aids, and limited opportunity for practice.^16^^-^^18^ Existing communication and SDM curricula utilize primarily lecture, small groups, role-play or simulation with a standardized patient (SP).^8^^,^^19^  Additionally, although some studies have demonstrated improvement in SDM skills compared to baseline or compared to a control group these studies have relied on one source for feedback such as either the student, a faculty member or a SP. We are not aware of existing curricula that evaluate the effectiveness of trainee SDM communication strategies utilizing multisource feedback and how often the result of their SDM discussions led to a common understanding with the patient of the agreed treatment plan. Assessing the effectiveness of SDM is important as the final goal of the communication strategy is to have an agreed upon treatment choice.

We designed an SDM curriculum for post-clerkship medical students including an SP simulation case depicting chronic sinusitis. The educational goals were to 1) lead a shared decision-making discussion with the patient regarding the two options for treatment (antibiotics vs. supportive measures); and 2) jointly decide which treatment plan the patient will follow. Students were provided multisource feedback from SP and faculty in addition to their self-assessment. We specifically selected a clinical scenario with no “right answer” to promote students’ engagement in shared decision-making.  Given that simulation is a high resource intervention, we sought to determine whether the multisource feedback provided additional insight for learners or if students’ self-assessment was sufficient, negating the need for faculty and/or SP feedback.

The aim of this case study is two-fold: first, to compare feedback from faculty, SPs and students regarding SDM communication skills using a standard rubric; second, to assess the effectiveness of the SDM discussion regarding whether the final shared-decision at the end of the visit was understood by both SP and student alike. We hypothesized that students would have different self-assessment scores compared to SP and faculty, indicating a need for multisource feedback and that some student-SP pairs would have differing conclusions about which treatment decision was supposedly decided.

## Methods

### Study Design

An observational case study design was chosen to examine the outcomes of a novel SDM curriculum designed for post-clerkship third-year medical students. The SP case was developed in response to a curricular need for SDM communications training and evaluation. All students received the same lectures and participated in the SDM case as part of their required curriculum thus there was no control group.

### Setting

Vanderbilt University School of Medicine is a four-year graduate medical school in Nashville, Tennessee, USA. Medical students who matriculate complete basic science courses their first year followed by six core clinical clerkships the second year. During the second-year clerkships, students learn the basics of clinical medicine including history-taking and physical exam skills. At the start of third year, all medical students must complete an Advanced Communication course training. The primary goal of the week-long Advanced Communications course is to help students develop and refine their patient-centered communication and interviewing skills and overcome common communication barriers. This course is part of a larger four-year longitudinal Foundations of Healthcare Delivery (FHD) course, which is required for all medical students as part of their MD degree program. Prior to the Advanced Communication course, medical students have completed lectures, small groups and assignments about patient-centered care, health care teams, interprofessional roles and responsibilities, patient education, health coaching and behavioral health change.

We completed the SDM SP case at the Vanderbilt Center Experiential Learning and Assessment (CELA), a simulation center with clinic rooms equipped with cameras for remote observation. Each of the 12 clinic rooms are identical and include an exam table, a bedside sink, a chair, and a table with a desktop computer.

### Educational Intervention

The SDM SP case is designed to assess SDM communication skills among post-clerkship medical students. The SDM case involves an SP who is a middle-aged person who presents to a primary care clinic for evaluation of nasal drainage and facial pain lasting for eight days, causing significant discomfort, and resulting in three missed days of work. The SP is instructed to express frustration with the ongoing illness and therefore presents for assessment and discussion of treatment options. The two options for treatment are antibiotics or supportive (symptomatic) measures. Depending on the discussion, the student and SP may opt for either treatment option with the goal of a shared understanding of the selected treatment at the end of the encounter. SPs are instructed to provide the following cues during the simulation: 1) display of anxiety and concern about losing job (empathy cue); 2) inquire about whether medications contain pork products (due to religious or dietary restrictions); and 3) request for explanation/definition in lay terms of one medical term.

In preparation for this SP encounter, students read “Shared Decision Making: A Model for Clinical Practice” by Elwyn and colleagues which includes a rationale for SDM in addition to a three-step model for clinical practice, described as “choice talk, option talk, and decision talk.” ^6^  The “choice talk” step involves letting patients know that there are reasonable options while the “option talk” step provides more details on the options available. The final step, “decision talk”, involves both support while considering the options and deciding which option is best. Additionally, students attended a large group didactic about the goals and proper techniques for SDM.

Students rotated through the simulation in groups of 12 based on the capacity of the simulation center. Simulation center staff oriented each group of students to the 25-minute-long encounter and asked students to review door notes. Students were instructed to complete the following three tasks: 1) using effective patient-centered communication techniques, lead a shared-decision making discussion with the patient regarding the two options for treatment (antibiotics vs. supportive measures); 2) jointly decide which treatment plan the patient will follow; and 3) once the treatment plan is selected, provide instructions to the patient on how to correctly take their medication(s) and confirm understanding. Students were to focus on discussing treatment options rather than history taking or exam: either antibiotics (amoxicillin/clavulanic acid) or supportive care (flunisolide nasal spray). It also summarized the clinical details for the case, noting that the patient is otherwise healthy, has had 8 days of facial pain with green drainage like prior episodes of sinusitis and has not tried any other medications for symptomatic relief. Further, the door notes included a summary of evidence-based practice guidelines for sinusitis with associated reference hyperlinks to the Infectious Disease Society of America (IDSA) Guidelines and the American College of Physicians (ACP) Choosing wisely Campaign.^20^^,^^21^ Both references guide clinicians that acute sinusitis with this presentation can reasonably be treated with either treatment option.  This information provided students with the requisite medical knowledge to complete the encounter and focus on communication strategies rather than diagnosis or treatment selection.

The encounter between student and SP lasted 25 minutes with a “2-minute remaining” verbal cue. Students could use any electronic resource during the encounter. Immediately following completion of simulation, students and SPs completed the same assessment rubric (see Table 1 in the Appendix). Each group of 12 students immediately met with simulation facilitators to debrief. The debrief session was to let students review individual written SP feedback and compare it with their self-assessment. At that time, the group reflected on their individual performance and any discrepancies between SP and self-assessment. The facilitator asked students general reflection questions such as “What did you find easy and challenging about this patient encounter?” and “Did you and the SP agree on the ultimate treatment decision? If not, what skills could you have utilized?” The facilitator’s role was to facilitate the debrief but they did not provide individual student feedback.

After the simulation was completed for all students, a faculty member with expertise in effective communication strategies asynchronously reviewed all recorded videos of student performance, provided students with written individualized formative feedback, and completed the same rubric.

### Participants

#### Medical Students

All post-clerkship medical students entering their third year of medical school at Vanderbilt University School of Medicine (VUSM) during 2016-2017 and 2017-2018 academic years (n=120) participated in this mandatory course and were thus included in the study. VUSM students are recruited primarily from the continental United States and represent diverse backgrounds. Specific demographic identifiers were not collected as part of this study given that the primary data source was from course assignments and feedback collected as part of the required course. This study utilized historical data for students that have graduated in the intervening time period. Because this study was initiated after course grades were finalized and students’ degrees granted, the Vanderbilt University Medical Center Institutional Review Board (IRB) granted ethical exemption for this project.  Furthermore, the dataset was de-identified and analyzed in aggregate. Therefore, the IRB, deemed the research exempted, or in other words, “not likely to adversely impact students' opportunity to learn required educational content” in accordance with existing ethics board policies. Given this exempt status, no participant consent was required.

#### Standardized Patients

Experienced simulation experts at the Vanderbilt Center for Experiential Learning and Assessment (CELA) maintain a pool of trained SPs with previous experience in acting and theatre. SPs for this case were recruited from this pool and received training on the case materials by specialized SP trainers to ensure quality control. SPs were trained on the evaluation rubric before the encounter.

#### Faculty Evaluator

The faculty evaluator was a faculty member from the Vanderbilt Center for Professionalism and Patient Advocacy (CPPA) with extensive experience in communications and professionalism training and was the course director for the Advanced Communications course.

#### Simulation Facilitator

For each group of 12 students, a simulation facilitator (faculty or resident trainee) synchronously observed aspects of each students’ interaction, requiring them to simultaneously monitor multiple encounters. Facilitators were selected faculty or staff from the Vanderbilt CPPA trained in providing feedback on communication methods. In some instances, resident trainees and other School of Medicine faculty members volunteered as facilitators when CPPA faculty were unavailable. These volunteer facilitators were oriented to the session's purpose and provided written session materials including suggested debrief discussion questions.

### Data Collection

#### Assessment Rubric Development

The assessment rubric was developed by a faculty member from Vanderbilt CPPA faculty with expertise in patient-centered communication strategies and was based on core principles of SDM transformed into observable behaviors.^6^ The assessment included 9 items (see Table 1 in the Appendix). Items 1 through 6 (maximum 30 points) assessed SDM skills; items 7 and 8 (maximum 10 points) assessed empathy, each using a 5-point Likert scale with behavioral anchors. The SDM skills included items such as describing treatment options without medical jargon, asking the patient his view about the proposed treatment, teach-back, and responding clearly to patient questions. Empathy items include demonstrating empathy and respect.  The last item was a multiple-choice question identifying which treatment option was collaboratively chosen at the close of the encounter. The assessment rubric was intentionally designed to be short and easily completed in real-time since SPs and students completed the assessment rubric immediately following the encounter and both were provided to students while the encounter was fresh in their minds.

#### Data Collection and Storage

The student and SP completed the assessment rubric immediately following the simulation as described above. After the simulation, the faculty evaluator reviewed all recorded videos of student performance and completed the rubric. The faculty completed these asynchronously for all students. All assessment rubrics were collected and managed using REDCap electronic data capture tools hosted at Vanderbilt University Medical Center.^22^^,^^23^  REDCap (Research Electronic Data Capture) is a secure, web-based software platform designed to support data capture for research studies, providing 1) an intuitive interface for validated data capture; 2) audit trails for tracking data manipulation and export procedures; 3) automated export procedures for seamless data downloads to common statistical packages; and 4) procedures for data integration and interoperability with external sources.

### Data Analysis

For purposes of this study, we used matched assessment rubrics from student, SP, and faculty to (1) determine concordance between students’ self-assessment, SP assessment, and faculty assessment of skills in SDM and empathy; and (2) to determine efficacy of communication as defined by rate of student-SP agreement on final treatment choice. We additionally sought to determine if higher SDM and empathy scores predicted treatment agreement between student and SP.  Assessment data was input into Microsoft Excel by authors V.I. and C.T. and stored securely with access limited to course directors and research personnel. We conducted this study after all participating students had graduated to minimize risk. We de-identified all files, assigning each student a number. Mean student scores on SDM and empathy scales were compared with SP and faculty assessments on identical scales using paired t-tests. Rate of student-SP agreement is expressed as percent of pairs reporting internally consistent treatment choices on matched rubrics. We used univariate logistic regression to assess whether performance on the SDM or empathy scales predicted agreement between student and SP on treatment plan. All statistical testing was two-sided at a significance level of 0.05. All analyses were conducted in a de-identified and aggregate fashion using STATA 12.1 statistical software (StataCorp, College Station, TX). There was no missing data.

## Results

Overall ratings of SDM and empathy skills among students (n = 120), SPs, and faculty are reflected in Table 2 in the Appendix.   On the SDM sub-scale, there was a significant difference between students self-rated SDM skills (M = 22.6, SD = 3.1) and SPs assessment of student SDM (M = 23.4, SD = 3.6), with students rating their SDM skills lower than SPs, t_(119)_ = 2.25, p = .027. There was no significant difference between students self-rated SDM and faculty ratings of SDM (M = 22.7, SD = 3.5), t_(119)_ = 0.46, p = .645.  On the empathy sub-scale, there was a significant difference between students self-rated empathy (M = 8.5, SD = 1.1) and SPs assessment of student empathy (M = 8.0, SD = 1.5), with students rating their empathy higher than SPs, t_(119)_ = 3.43, p < .001. There was no significant difference between students self-rated empathy and faculty ratings of student empathy, (M = 8.3, SD = 1.7), t_(119)_ = 1.40, p = .164.

A majority (86%, 103 of 120) of student/SP pairs agreed on the selected treatment plan. Of the 14% who did not agree, the faculty observer agreed more often with the student’s selection (10/17) than the SP and there was no discernible pattern of discordant selections. Finally, in univariate logistic regression models, neither performance on the SDM nor empathy subscale was predictive of treatment disagreement (see Table 3 in the Appendix). Treatment disagreement was slightly more frequent among students who were assessed by the faculty as having lower empathy (OR = 1.23, 0.93 – 1.62, p = .152) but did not reach statistical significance.

Although no specific qualitative data was collected from the debrief sessions, simulation facilitators reported that students generally expressed gratitude for the opportunity to practice a new set of communication skills which they found the simulation helpful and realistic. Unique features about this case allowed students to reflect on their approach to patient inquiries and the degree of their empathetic responses. Some students were surprised that the SP rated their communication skills lower than their perceived skills, which prompted insightful discussion about the importance of patient perspective and clear communication. Students were appropriately challenged by this case and simultaneously inspired to continue to improve their SDM communication skills.

## Discussion

The Shared Decision-Making (SDM) SP case represents a novel, interactive didactic designed to provide medical students with a low-stakes simulated environment to practice SDM communication skills. We found that multisource assessment provides additional differing feedback when practicing SDM and that in a subset of student-SP pairs there was disagreement regarding the perceived final treatment choice that was supposedly collaboratively agreed upon, emphasizing the need for ongoing SDM practice and training.

The case is intentionally designed to simulate a common clinical complaint (sinusitis) and represents a situation where two treatment options are equally reasonable. We found that this was an important aspect of the case design because it allowed early medical learners to focus on the core SDM communication skills rather than taking histories or worry about explaining the clinical aspects of the case to the SP.  Although SDM is a critical skill for all medical professionals, introducing this skill to early medical learners allows students time to seek opportunities for deliberate practice and feedback prior to graduation and independent practice.  Furthermore, early clinical learners are still developing their clinical reasoning. Introducing patient-centered communication strategies such as SDM into their developing clinical reasoning, promotes incorporation of patient preference into the final decisions. Understanding how to elicit patient preference, effectively explain treatment options, and collectively decide on a treatment plan is imperative to effective clinical reasoning. ^24^ Expressions of empathy are an essential component to effectively demonstrating patient-centered communication techniques. ^4^^,^^6^ This SP encounter therefore intentionally included and assessed triggers for empathy.

Comparing the feedback from student, SP and faculty helped us understand that there are distinct differences in perception of SDM skills and empathetic expression across the groups. Variability in student self-scoring compared to SP assessment underscores that additional communication training may be necessary to ensure that students can effectively use SDM and identify good SDM practices. Faculty feedback for all students was done by a single individual with expertise in patient-centered communication which was a significant strength of the study. While SP and medical student assessments differed significantly, it was interesting that SP and faculty assessments also differed in a similar pattern. SPs trended toward higher SDM scores and lower empathy scores compared to both students and faculty. One interpretation would be that the faculty may be especially stringent on assessing student SDM performance given their familiarity and expertise with SDM. SPs may have expected a more genuine or prolonged display of empathy from students than faculty did. Prior research has demonstrated mixed results on inter-rater reliability between SP and instructors, with some authors, such as Teker and Odabaşı, finding that SPs rate communication skills more favorably than faculty.^25^ Meanwhile, Talwalkar and colleagues reported improved reliability for advanced communication skills.^26^ This variability may be related to differing expectations among groups of assessors and is surely confounded by bias. Although SP feedback may not be an accurate substitute for faculty feedback, our case study highlights the value of multisource feedback to support trainee skill performance.

The efficacy of communication was assessed by a percentage of student-SP pairs that reported consistent, supposedly collectively agreed upon treatment choice at the end of the visit. The fact that in 14% of cases, a student and SP could complete the encounter with opposite conclusions about what treatment strategy they had decided on proves that legitimate communication gaps remained between student intention and patient understanding. This gap highlights the need for ongoing SDM practice to improve efficacy of communication.

Although feasibility and resource utilization were not the primary aims of this study, we found that the simulation was simple, realistic, adaptable, and promoted student self-reflection. For example, the case materials are simple and did not require lengthy SP training sessions or preparation. In fact, it could reasonably be adapted as a role play scenario or virtual encounter in settings with fewer simulation resources. Given the nature of the interaction, this case could also be repeated longitudinally to assess skill acquisition over time. This type of simulation supports student reflective practice by providing multisource feedback, most critically from the SP.

Our study is not without limitations. Students were not provided substantial training on SDM beforehand, which may have limited their ability to successfully demonstrate SDM communication techniques. Alternatively, antecedent communication training on patient education and behavioral health modifications in previous courses may have primed learners for relative success with SDM without extensive training ahead of the simulation. Given that students have few authentic training experiences and opportunities to apply SDM in clinical encounters, this SDM case was designed for formative learning rather than high-stakes assessment. Likewise, SPs were not trained in SDM communication techniques, and no formal validation of the rubric was performed beforehand. Differences between individual SPs could have accounted for some variability, however we did not collect SP-level data required to calculate inter-rater reliability scores. Finally, this case was implemented at a single academic institution which limits generalizability. Despite these limitations, this SDM case has continued to be used in our curriculum since 2016 and has been a consistent highlight of the communication curriculum on student course evaluations. Students particularly value being able to practice implementing these skills in an authentic manner and receive multi-source feedback on their performance, which allows them to set personalized learning goals.

Future research could focus on expanding validity evidence for this rubric, an important component of which would be to correlate our findings with other measures of patient-centered communication or at a variety of time points during medical school training. Educational interventions have been shown to improve ratings of patient-centered communication.^27^ Variability and lack of validity data among communication assessment tools continues to limit comparison across instruments.^3^^,^^28^ Still, certain behaviors are consistently correlated with higher overall ratings of communication including empathy, reassurance, summarizing, and clarification and additional research could include established measures of excellence in communication including the efficacy of decision making as measured in this study.^2^

## Conclusion

In summary, this SDM case is a novel simulation designed as a formative experiential learning exercise for medical trainees to practice patient-centered interviewing techniques, specifically SDM. This case depicts a realistic and common clinical scenario with two equally valid treatment choices. Use of a universal assessment rubric allows for multi-source feedback which students found helpful in promoting insight and reflection on their communication skills to guide and motivate their skill development prior to graduation and independent practice. Assessment of final treatment decision with discovery of significant treatment disagreement further highlighted the importance of improving SDM skills.

### Acknowledgements

The authors would like to acknowledge the contribution of Dr. Lynn Webb, whose expertise in developing this case and rubric as well as in providing feedback to this group of students was invaluable to not only this study, but their learning and development.

### Conflict of Interest

The authors declare that they have no conflicts of interest.
