# Supplementary material for: Assessing shared decision-making: a case study of third-year medical student standardized patient encounters
Source: Int J Med Educ. 2025 Jan 22;16:1–10. doi: 10.5116/ijme.676f.d093 (PMC12700522; doi:10.5116/ijme.676f.d093)
Supplement: Supplementary file 1 — Appendix [file ijme-16-1-S1.pdf]

Appendix

Table 1. Assessment Rubric

Item	Likert Scale					Sub scores
	1	2	3	4	5	
1. Confirms/ explains the main problems being addressed	Does not review problems before reviewing treatment.	Between 1 and 3	Describes key problems; provides little opportunity for patient input	Between 3 and 5	Thoroughly reviews problems in language easy to understand; confirms that patient agrees with problems outlined; asks about any other problems	SDM ITEMS (30 points)
2. Describes treatment options without medical jargon and/or explains medical terms	Uses difficult medical terms with no explanation	Between 1 and 3	Uses some jargon; allows patient to ask for clarification	Between 3 and 5	Uses plain language when describing each option or uses a medical term, then follows with easily understood description	
3. Describes evidence for and against each option presented	Describes options with no explanation for why it should/should not be considered	Between 1 and 3	Offers cursory evidence for or against options. Uses default terms such as "best practice"	Between 3 and 5	For each option, describes the potential benefits and risks to consider	
4. Asks patient his/her view about the treatment that is being proposed	Does not invite patient to state preference/opinion/expectations about the treatment plan	Between 1 and 3	Uses wording such as "Is that okay?" or "Do you understand?"	Between 3 and 5	Asks the patient what he/she thinks about the treatment options; re-examines treatment plan based upon patient's responses	
5. Demonstrates teach back by asking patient to explain something about the plan	Does not ask patient to explain or describe any part of treatment plan to unsure understanding	Between 1 and 3	Simply asks if patient understands the plan without using teach back technique	Between 3 and 5	Asks the patient to describe in detail at least one part of the treatment plan; confirms accuracy/clarified	
6. Responds to questions in a manner clearly understood by patient	Uses mostly medical terms without explanation	Between 1 and 3	Responds to questions but has difficulty using language easily understood by the patient	Between 3 and 5	Consistently uses terms that patient understands or provides explanation when using a specific medical term	
7. Demonstrates empathy in response to appropriate cues	No evidence of empathy throughout the entire encounter	Between 1 and 3	Listens to patient, maintains eye contact, minimal verbal response.	Between 3 and 5	Responds appropriately to patient cue for empathy; uses empathy at other times as appropriate; may use probing "tell me more"	EMPATHY ITEMS (10 points)
8. Demonstrates respect; not condescending or judgmental	Patient feels judged or disrespected at least one time	Between 1 and 3	Demonstrates respect (not interrupting, addressing patient appropriately)	Between 3 and 5	Highly respectful throughout interview; patient feels an equal partner throughout the encounter	
9. Which treatment alternative was selected? (select one)	Antibiotics	--	Supportive measures	--	No definite decision	Treatment Choice

Table 2. Summary of raw performance on shared decision-making (SDM) and empathy subscales (VUSM 2016-2017) n=120

Variable	SDM subscale (max 30)	Empathy subscale (max 10)
	mean, SD	mean, SD
Student	22.6 (3.1)	8.5 (1.1)
SP	23.4 (3.6)	8.0 (1.5)
Faculty	22.7 (3.5)	8.3 (1.7)

Table 3. Performance by students on SDM and empathy subscales and treatment agreement

VUSM 2016-2017, n=120

Variable	Student-SP Pairs in agreement (n=103)	Student-SP Pairs in disagreement (n=17)	OR crude (95% CI)	p-value*
Student SDM assessment	22.5	22.8	0.97 (0.82 – 1.16)	.803
SP SDM assessment	23.3	24.0	0.95 (0.82 – 1.10)	.481
Faculty SDM assessment	22.9	22.9	1.00 (0.86 – 1.16)	.957
Student empathy assessment	8.54	8.41	1.12 (0.70 – 1.81)	.634
SP empathy assessment	8.03	7.76	1.13 (0.80 – 1.59)	.491
Faculty empathy assessment	8.41	7.76	1.23 (0.93 – 1.62)	.152

*Univariate logistic regression
